# Supplementary material for: Deep-Neural-Networks-Based Data-Driven Methods for Characterizing the Mechanical Behavior of Hydroxyl-Terminated Polyether Propellants
Source: Polymers (Basel). 2025 Feb 28;17(5):660. doi: 10.3390/polym17050660 (PMC11902718; doi:10.3390/polym17050660)
Supplement: Supplementary file 1 [file polymers-17-00660-s001.zip › polymers-3463770-supplementary.pdf]

Supporting Information

Deep neural networks based data-driven methods for characterizing the mechanical behavior of hydroxyl-terminated polyether propellants

Ruohan Han[1], Xiaolong Fu[1,]*, Bei Qu[1], La Shi[1] and Yuhang Liu[2]

[1]Xi'an Modern Chemistry Research Institute, Xi'an, Shaanxi 710065, China

[2]Department of Geotechnical Engineering, Tongji University, Shanghai 200092, China

Corresponding Author E-mail: fuxiaolong204@163.com

1. LSTM model architecture

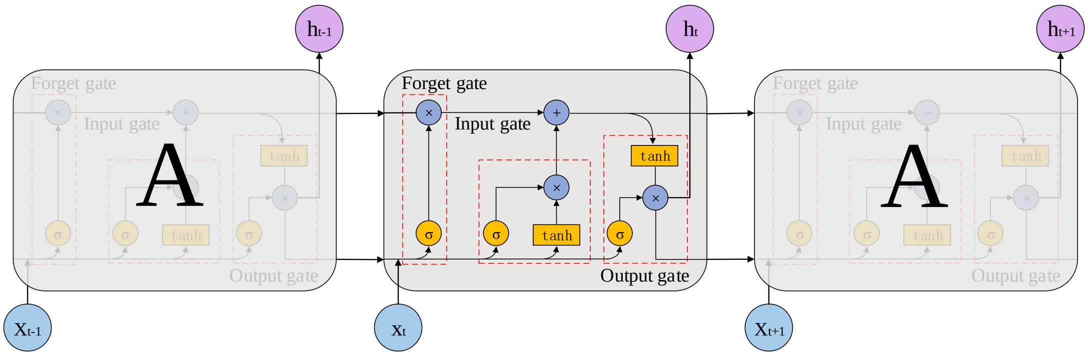

FIGURE S1 LSTM model architecture.

2. Variation of RMSE with the number of data points on each stress-strain curve from modeling HTPE propellant using the FFNN approach.

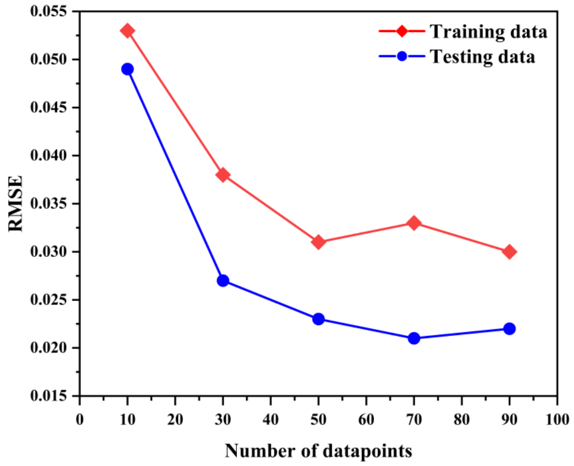

FIGURE S2 Variation of RMSE with the number of data points on each stress-strain curve from modeling HTPE propellant using the FFNN approach.
